# Associations Between Hepatic and Pancreatic Steatosis with Lumbar Spinal Bone Marrow Fat: A Single-Center Magnetic Resonance Imaging Study

**DOI:** 10.5152/tjg.2023.22225

**Published:** 2023-06-01

**Authors:** Akın Abbasoğlu, Musturay Karçaaltıncaba, Ali Devrim Karaosmanoğlu, Mustafa Nasuh Özmen, Deniz Akata, İlkay S. İdilman

**Affiliations:** 1 Department of Radiology, Hacettepe University Faculty of Medicine, Liver Imaging Team, Ankara, Turkey

**Keywords:** Liver, pancreas, lumbar, steatosis, MRI-PDFF

## Abstract

**Background:**

To evaluate the associations between hepatic, pancreatic steatosis, and lumbar spinal bone marrow fat determined by magnetic resonance imaging-proton density fat fraction in patients with no known or suspected liver disease.

**Methods:**

A total of 200 patients who were referred to our radiology department for upper abdominal magnetic resonance imaging between November 2015 and November 2017 were included in this study. All patients underwent a magnetic resonance imaging-proton density fat fraction on a 1.5-T magnetic resonance imaging system.

**Results:**

The mean liver, pancreas, and lumbar magnetic resonance imaging-proton density fat fraction were 7.52 ± 4.82%, 5.25 ± 5.44%, and 46.85 ± 10.38% in the study population. There were significant correlations between liver and pancreas (*r*_s_ = 0.180, *P* = .036), liver and lumbar (*r*_s_ = 0.317, *P* < .001), and pancreas and lumbar magnetic resonance imaging-proton density fat fraction (*r*_s_ = 0.215, *P* = .012) in female patients. A weak correlation was observed between liver and lumbar magnetic resonance imaging-proton density fat fraction (*r*_s_ = 0.174, *P* = .014) in the total population. The prevalence of hepatic and pancreatic steatosis was 42.5% and 29%, respectively. The prevalence of pancreatic steatosis (42.9% vs. 22.8%, *P* = .004) was higher in male patients compared to female patients. In subgroup analysis, in patients with hepatic steatosis, there were higher pancreas magnetic resonance imaging-proton density fat fraction (6.07 ± 6.42% vs. 4.66 ± 4.53%,* P* = .036) and lumbar magnetic resonance imaging-proton density fat fraction (48.81 ± 10.01% vs. 45.40 ± 10.46%,* P* =.029) compared to patients without hepatic steatosis. In patients with pancreatic steatosis, there were higher liver (9.07 ± 6.08 vs. 6.87 ± 4.06,* P* = .009) and lumbar magnetic resonance imaging-proton density fat fraction (49.31 ± 9.13% vs.45.83 ± 10.76%,* P* = .032) in comparison with patients without pancreatic steatosis.

**Conclusion:**

Based on the results of the present study, fat accumulation in liver, pancreas, and lumbar vertebra have associations with more evident in females.

**Main Points**

- There is a correlation between fat accumulation in liver, pancreas, and lumbar vertebra which is more evident in female patients.
- The hepatic and pancreatic steatosis prevalence is observed as 42.5% and 29% in Turkish population which were evaluated for different abdominal pathologies.
- The associations between fat accumulation in liver, pancreas, and lumbar vertebra can be evaluated with magnetic resonance imaging-proton density fat fraction as well as hepatic and pancreatic steatosis prevalence.

## INTRODUCTION

Nonalcoholic fatty liver disease (NAFLD) is the excessive triglyceride accumulation in hepatocytes with an increasing prevalence correlated with obesity.^1,2^ It is estimated that NAFLD would be the main indication for liver transplantation in the future.^3^ The gold standard for the diagnosis of NAFLD is liver biopsy. However, it has potential complications such as bleeding and perforation and is prone to sampling errors as it represents approximately 1/50 000th of the liver.^4,5^ Therefore, non-invasive diagnostic tools such as imaging modalities are increasingly used for diagnosis and follow-up evaluation of NAFLD patients.

A recent imaging method, magnetic resonance imaging-proton density fat fraction (MRI-PDFF), was shown to be an accurate tool for quantification of liver fat in NAFLD.^6,7^ Beside evaluation of hepatic steatosis, MRI-PDFF can also be used for fat quantification of different organs such as pancreas and bone marrow.^8^ Some studies evaluated the relationship between hepatic, pancreatic, and lumbar bone marrow fat in patients with NAFLD with MRI-PDFF.^8,9^ Idilman et al^8^ observed a good correlation between pancreas and vertebral body MRI-PDFF in NAFLD patients and also showed that both of them are higher in diabetic patients. Patel et al^9^ also showed an association between pancreatic fat and hepatic steatosis in NAFLD patients. Pancreatic fat content determined by MRI-PDFF was also studied in patients with NAFLD and healthy controls and it was observed that pancreatic fat was higher in patients with NAFLD.^10^

In this study, we aimed to evaluate hepatic and pancreatic steatosis prevalence and associations between hepatic, pancreatic, and lumbar spinal bone marrow fat determined by MRI-PDFF in general population.

## MATERIALS AND METHODS

We included a total of 200 patients which were referred to radiology department for upper abdominal MRI between November 2015 and November 2017. This retrospective study was approved by the institutional review board of Hacettepe University.

### Patients

A total of 856 abdominal MR examinations were performed between November 2015 and November 2017 in our radiology department for several indications. Oncology patients (n = 422), patients with chronic liver disease and pediatric population (n = 38) were excluded. The patients who were referred to MRI with a clinical suspect of NAFLD or who has a previous NAFLD diagnosis were also excluded (n = 178). Additionally, 18 patients were excluded as ineligible MRI-PDFF images. A total of 200 patients were included in the study population. The main indications for imaging was liver lesions, such as hemangiomas, focal nodular hyperplasia, and cysts (n = 73) in the study population. The remaining patients were examined for pathologies concerning different organs such as gall bladder or biliary diseases (n = 34), pancreatic lesions (n = 21), renal lesions (n = 18), adrenal lesions (n = 18), and splenic lesions (n = 6). The remaining 30 patients were examined for abdominal pain, gastrointestinal tract diseases, gynecologic diseases, and hernias. The institutional review board waived the informed consent as a result of retrospective nature of the study.

### Magnetic Resonance Imaging

All patients underwent an MRI-PDFF on a 1.5-T MR imaging system (Achieva, Philips Medical Systems). An 8-channel phased-array body coil was used for this acquisition and all patients were examined in the supine position. All patients underwent mDIXON Quant sequence with the following parameters: repetition time, 5.3 ms; field of view, 35-40 cm; matrix, 224 × 99; flip angle, 5°; section thickness, 5 mm; and a single 3-dimensional image with 67 sections. The sequence was acquired in a breath-hold time (25 seconds) in all patients.

Image processing: By using a workstation (HP Z 440), a radiologist (D.A.) placed a round of interest (ROI) of 1 cm^2^ at all 8 liver segments, an ROI of 0.5-1 cm^2^ at pancreatic head, body, and tail and an ROI of 1.5 cm^2^ at first 3 lumbar spines on the fat fraction images calculated from the mDIXON Quant sequence. These ROI sizes were chosen according to interested organs’ area. All ROIs were placed within the interested tissue with avoiding focal lesions, major vessels, ducts, and collecting systems and by being sure that ROI is surrounded by the interested tissue. Mean liver MRI-PDFF, pancreas MRI-PDFF, and lumbar MRI-PDFF were calculated.

The following threshold values for liver MRI-PDFF were used: PDFF of 6.4% or less, grade 0 (no fat content); PDFF greater than 6.4%, grade 1 (mild fat content); PDFF greater than 17.5%, grade 2 (moderate fat content); and PDFF greater than 22.1%, grade 3 (high fat content).^6^ According to the Singh et al^11^ we grouped the patients as presence of pancreatic steatosis or not with a cut-off value of 6.2%.

### Statistical Analyses

Data were summarized as mean ± SD or median (range) for continuous variables depending on the distributional properties of the data. Normality of the variables was tested by Kolmogorov–Smirnov test. Student’s *t*-test or Mann–Whitney *U*-test was used to assess differences in continuous variables between groups. One-way ANOVA or Kruskal–Wallis test was used to assess differences in continuous variables between more than 2 groups. Categorical variables were evaluated by Pearson’s Chi-square test. The degree of association between continuous and/or ordinal variables was calculated by using the Pearson correlation coefficient or Spearman’s rho. Correlation coefficient (*ρ*) > 0.7 was considered strong, 0.4-0.7 was considered as moderate and lower than 0.4 was considered weak.^12^ For all the tests, a 2-tailed *P*-value of less than .05 was considered as statistically significant. All statistical analysis was performed on the Statistical Package for Social Sciences (SPSS) version 22.0 (IBM Corp.; Armonk, NY, USA).

## RESULTS

A total of 200 individuals (M/F = 64/136) were included in the study. Mean age of the study population was 48.6 ± 12.3 years (median 49 years; range, 21-76 years). The mean ± SD of serum AST, ALT, GGT, and total bilirubin levels were 26.4 ± 26.6 U/L, 27.4 ± 31.8 U/L, 47 ± 73.6 U/L, and 0.7 ± 0.4 mg/dL, respectively. The characteristics of all patients are shown in Table 1.

Mean liver MRI-PDFF of segment 1, 2, 3, 4, 5, 6, 7, and 8 was 7.40 ± 4.82%, 7.17 ± 4.64%, 7.16 ± 4.71%, 7.50 ± 4.90%, 7.64 ± 4.93%, 7.84 ± 5.57%, 7.70 ± 4.98%, and 7.76 ± 5.03%, respectively. There was no significant difference among calculated PDFFs with respect to different liver segments (*P* = .524). Mean liver MRI-PDFF of right and left lobe was 7.73 ± 4.98% and 7.28 ± 4.71%, respectively, with no statistically significant difference (*P* = .218). Mean liver MRI-PDFF was 7.52 ± 4.82%.

**Figure 1.**
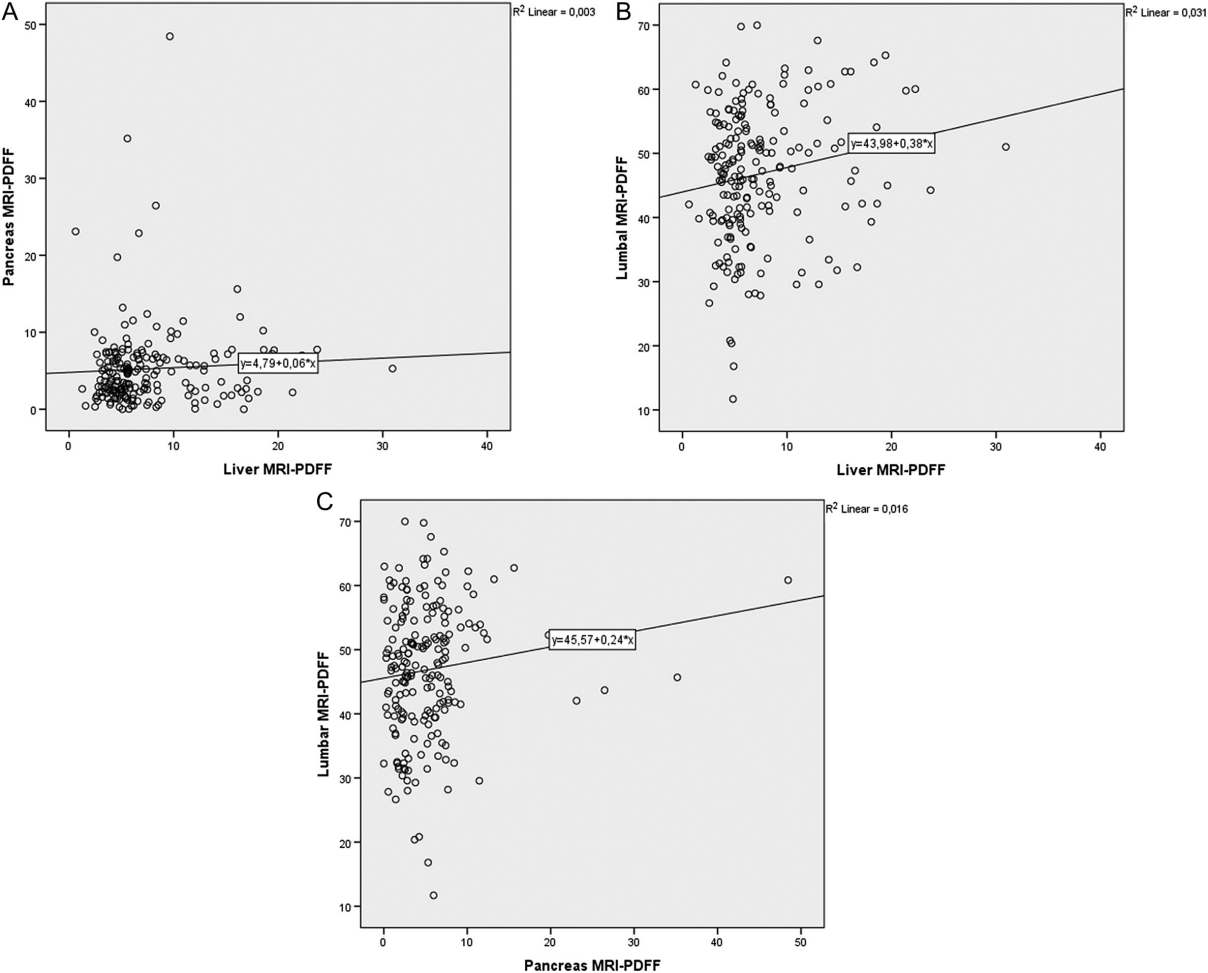
The correlations between liver and pancreas (A), liver and lumbar spinal bone marrow (B), and pancreas and lumbar spinal bone marrow MRI-PDFF (C) in general population.

Mean pancreas MRI-PDFF of the head, body, and the tail was 5.22 ± 5.67%, 5.33 ± 5.33% and 5.20 ± 5.46%, respectively, with no statistically significant difference (*P* = .965). Mean pancreas MRI-PDFF was observed as 5.25 ± 5.44%. Mean lumbar MRI-PDFF of L1, L2, and L3 was 46.53 ± 10.56%, 47.10 ± 10.59%, and 46.92 ± 10.40% with no statistically significant difference (*P* = .936). Mean lumbar MRI-PDFF was observed as 46.85 ± 10.38% (Table 2). There was a weak but statistically significant correlation between liver and lumbar MRI-PDFF (*r*_s_ = 0.174, *P* = .014). However, no correlation was observed between liver and pancreas MRI-PDFF (*r*_s_ = 0.125, *P* = .078) and pancreas and lumbar MRI-PDFF (*r*_s_ = 0.131, *P* = .066) in the general population (Figure 1). There was a moderate correlation between age and lumbar MRI-PDFF (*r* = 0.559, *P* < .001), weak but statistically significant correlation between age and liver MRI-PDFF (*r*_s_ = 0.215, *P* = .002) and age and pancreas MRI-PDFF (*r*_s_ = 0.191, *P* = .007).

**Figure 2.**
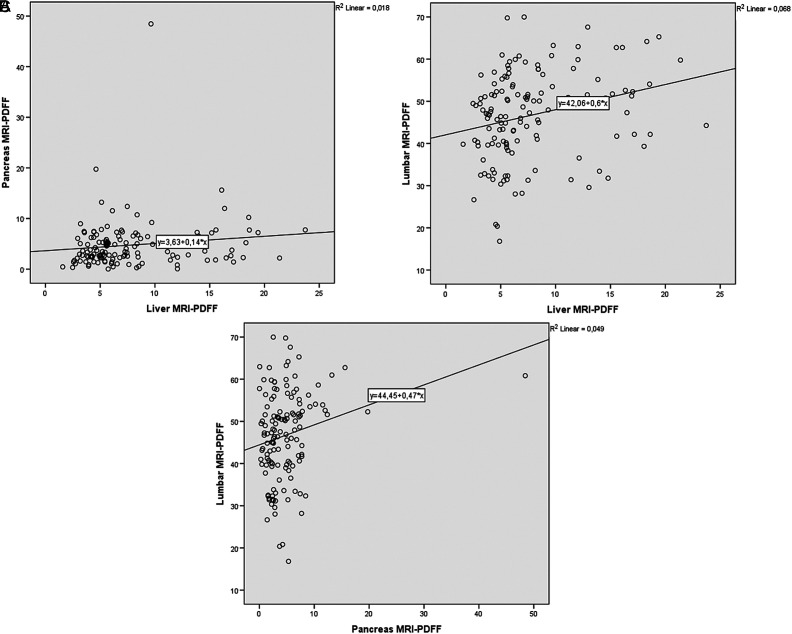
The correlations between liver and pancreas (A), liver and lumbar spinal bone marrow (B), and pancreas and lumbar spinal bone marrow MRI-PDFF (C) in female patients.

**Figure 3.**
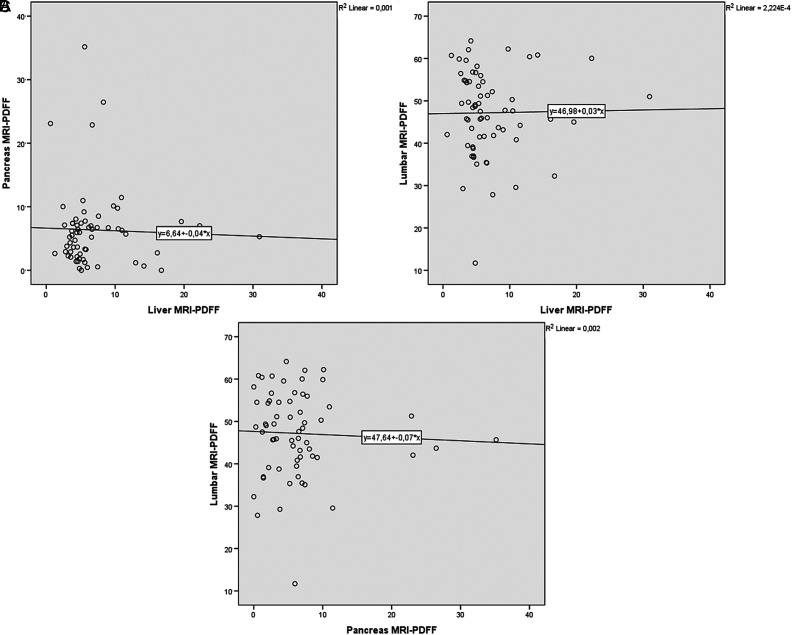
The correlations between liver and pancreas (A), liver and lumbar spinal bone marrow (B), and pancreas and lumbar spinal bone marrow MRI-PDFF (C) in male patients.

When we grouped the patients according to gender, we observed higher liver MRI-PDFF in female patients in comparison with male patients with no statistical significance (7.78 ± 4.63 vs. 6.98 ± 5.18, *P* = .072). However, mean pancreas MRI-PDFF was higher in male patients with statistical significance (6.35 ± 6.26 vs. 4.74 ± 4.95, *P* = .041). In subgroup analyses, there was a weak but statistically significant correlation between liver and pancreas MRI-PDFF (*r*_s_ = 0.180, *P* = .036), and liver and lumbar MRI-PDFF (*r*_s_ = 0.317, *P* < .001) and pancreas and lumbar MRI-PDFF (*r*_s_ = 0.215, *P* = .012) in female patients (Figure 2). However, there were no statistically significant correlation between liver and pancreas MRI-PDFF (*r*_s_ = 0.078, *P* = .543), liver and lumbar MRI-PDFF (*r*_s_ = –0.138, *P* = .278), and pancreas and lumbar MRI-PDFF (*r*_s_ = -0.045, *P* = .724) in male patients (Figure 3).

A total of 85 patients (42.5%) had hepatic steatosis. A total of 75 patients (37.5%) had grade 1 hepatic steatosis, 7 (3.5%) had grade 2 hepatic steatosis, and 3 (1.5%) had grade 3 hepatic steatosis according to the MRI-PDFF measurement. The prevalence of hepatic steatosis in female patients was slightly higher with no statistical significance (45.6% vs. 35.9%, *P* = .198). A total of 58 patients (29%) had pancreatic steatosis. The prevalence of pancreatic steatosis in male patients was significantly higher (42.9% vs. 22.8%, *P* = .004). The mean pancreas MRI-PDFF was 6.07 ± 6.42% in patients with hepatic steatosis and 4.66 ± 4.53% in patients with no steatosis and significantly higher in the hepatic steatosis group (*P* = .036). The mean lumbar vertebral MRI-PDFF was 48.81 ± 10.01% in patients with hepatic steatosis and 45.40 ± 10.46% in patients with no steatosis and significantly higher in the steatosis group (*P* = .029). Lumbar PDFF was statistically significantly higher in patients with pancreatic steatosis in comparison with no pancreatic steatosis (49.31 ± 9.13%, vs. 45.83 ± 10.76%, *P* = .032). Similarly, liver MRI-PDFF was significantly higher in patients with pancreatic steatosis (9.07 ± 6.08 vs. 6.87 ± 4.06 *P* = .009).

## DISCUSSION

In this study, there was no significant difference among liver segments and pancreatic regions in terms of MRI-PDFF. There was a weak correlation between liver and lumbar MRI-PDFF, whereas no correlation was observed between liver and pancreas MRI-PDFF and pancreas and lumbar MRI-PDFF in the general population. In the subgroup analyses, we observed significant correlations among liver, pancreas, and lumbar MRI-PDFFs in female patients. However, we did not observe significant correlations among the same parameters in male patients. In patients with hepatic steatosis, we observed higher MRI-PDFF values in pancreas and lumbar vertebrae. We also demonstrated a hepatic steatosis and pancreatic steatosis prevalence of 42.5% and 29%, respectively, in the Turkish population which were evaluated for different abdominal pathologies with MRI-PDFF. The hepatic steatosis prevalence was slightly higher in the female patients and pancreatic steatosis was significantly higher in male patients.

The NAFLD prevalence may differ according to different regions and countries of the world with a recently reported prevalence of 48.3% in Turkey.^13^ The difference in our prevalence may be related to inclusion criteria as we just included patients with no suspected liver disease including NAFLD. However, Değertekin et al^13^ included all patients applied to the check-up clinics after exclusion for liver diseases except NAFLD. Değertekin et al^13^ observed a higher prevalence in male patients (72.1% vs. 37.9%, *P* < .001) in contrast with our study which can be explained by higher mean age of our patient population. Kühn et al^14^ observed a very similar prevalence of fatty liver disease (42.2%) in a German population with a similar MRI technique. In accordance with Değertekin et al^13^, Kühn et al^14^ observed higher prevalence of fatty liver disease in male patients (50.9% vs. 34.7%).

The most common hepatic steatosis pattern is diffuse form followed by different heterogeneous hepatic steatosis patterns.^15^ The studies evaluating liver steatosis with MRI demonstrated conflicting results in terms of hepatic steatosis heterogeneity according to different liver segments and lobes.^7,16,17^ Bonekamp et al^16^ observed higher MRI-PDFF values in the right lobe in comparison with left lobe. In accordance with this study, Capitan et al^17^ observed higher liver fat content in the right lobe. In this study, the authors also observed heterogeneous hepatic steatosis in both lobes and in patients with and without hepatic steatosis. In our study, we did not observe a significant difference at both segmental and lobar levels. In accordance with the present study, Idilman et al^7^ also did not observe such a difference in a NAFLD patient population.

A recent study observed that measuring different regions of pancreatic MRI-PDFF has great variability in a population of NAFLD.^18^ It is also confirmed by the population-based study which included 1367 individuals and observed 4.6% pancreatic PDFF in the head, 4.9% in the body, and 3.9% in the tail.^19^ However, we did not observe such a difference in a population-based study which also includes patients without NAFLD in accordance with Patel et al’s^9^ and Idilman et al’s^8^ studies which included just NAFLD patients. In contrast with Kühn et al’s^14^ study, our population had higher pancreatic fat contents. In our study population, male patients had higher pancreas fat with statistically significant difference. We also observed higher pancreatic steatosis rate in male patients with statistical significance. In accordance with the present study, Kato et al^18^ observed that male gender is a risk factor for pancreatic steatosis.

There are studies evaluating relationship between liver and pancreatic fat with MRI-PDFF with conflicting results.^8-10,20-22^ Most of them demonstrated a significant correlation among them, whereas Idilman et al^8^ did not observe such a correlation. In accordance with Idilman et al’s^8^ study, we also did not demonstrate a correlation between liver and pancreas MRI-PDFF in the general population. However, in the subgroup analyses, we observed significant correlations among them in female patients. There was also a significant correlation between liver and lumbar MRI-PDFF in our general population in accordance with Idilman et al’s^8^ study. This correlation remained significant in the subgroup analyses of female patient population. The knowledge of this association is important as increase in liver fat may be related with osteoporotic bone fractures which was also shown in a recent meta-analyses.^23^ Interestingly, there is no correlation among liver, pancreas, and lumbar vertebra MRI-PDFFs in male patients which is not evaluated already in the literature. This difference can be a result of different fat metabolisms in male and female patients which should be evaluated with further studies.

There are some strengths and limitations in the current study. This is the first MRI-based study which evaluates the prevalence of hepatic steatosis and associations between hepatic, pancreatic steatosis, and lumbar spinal bone marrow fat determined by MRI-PDFF in patients referred to abdominal MRI with different indications. The main limitation is the relatively small sample size which is a result of our expanded exclusion criteria. Another limitation is the lack of clinical data of the patients such as diabetes status and metabolic syndrome which may also affect fat deposition in different organs. Extensive studies with larger sample size and clinical data will help understanding the fat metabolism and relationships between fat deposition in different organs.

In conclusion, fat accumulation in liver, pancreas, and lumbar vertebra have associations with more evident in female patients. We observed a hepatic and pancreatic steatosis prevalence of 42.5% and 29%, respectively, in the Turkish population which was evaluated for different abdominal pathologies with MRI-PDFF.

## Figures and Tables

**Table 1.** Characteristics of the Study Population

	Male (n = 64), mean ± SD	Female (n = 136), mean ± SD	*P*
Age			
48.56 ± 12.28	47.84 ± 12.51	48.92 ± 12.21	.565^*^
	49 (21-76)	49 (24-74)	50 (21-76)	
Serum AST level (n: 10-37 U/L)			
26.40 ± 26.55	26.44 ± 15.15	26.37 ± 30.92	.122^**^
	21 (13-267)	22 (14-94)	20 (13-267)	
Serum ALT level (n: 10-37 U/L)			
27.42 ± 31.81	28.47 ± 23.38	26.89 ± 35.41	.028^**^
	19.5 (4-292)	24 (9-169)	17 (4-292)	
Serum GGT level (n: 0-55 U/L)			
47.00 ± 73.57	49.06 ± 61.80	45.95 ± 79.14	.020^**^
	26 (9-613)	31 (12-360)	22 (9-613)	
Serum total bilirubin level (n: 0.2-1.3 mg/dL)			
0.70 ± 0.39	0.80 ± 0.38	0.65 ± 0.38	.005^**^
	0.59 (0.21-2.94)	0.68 (0.31-1.68)	0.56 (0.21-2.94)	
Platelet			
237.73 ± 66.79	219.60 ± 74.21	246.96 ± 60.98	.001^**^
	234.5 (98-526)	205.5 (98-526)	247 (134-478)	
Fasting glucose (n: 75-115 mg/dL)			
98.11 ± 24.79	97.63 ± 22.30	98.33 ± 26.02	.838^**^
	91 (58-229)	91.5 (79-199)	91 (58-229)	
Cholesterol (n: 120-200 mg/dL)			
220.09 ± 46.91	219.68 ± 45.15	220.30 ± 48.40	.986^**^
	212 (131-366)	217 (135-322)	212 (131-366)	
Triglyceride (n: 40-165 mg/dL)			
173.76 ± 118.63	168.77 ± 75.02	177.36 ± 143.10	.454^**^
	142 (44-753)	146 (60-337)	139.5 (44-753)	
HDL (n: 40-60 mg/dL)			
49.81 ± 11.79	45.05 ± 9.46	52.38 ± 12.23	.027^**^
	50 (32-82)	44.5 (32-66)	53 (33-82)	

Data are mean ± SD with median (range).* Mann-Whitney U-test,** Student’s t-test.

ALT, alanine aminotrasnferase; AST, aspartate aminotrasnferase; GGT, gamma glutamyl transpeptidase; HDL, high-density lipoprotein.

**Table 2.** MRI-PDFF Measurements of the Study Population

	Male (n = 64), mean ± SD	Female (n = 136), mean ± SD	*P*
Liver MRI-PDFF (%)			
7.52 ± 4.82	6.98 ± 5.18	7.78 ± 4.63	.073^**^
	5.65 (0.62-30.94)	5.34 (0.62-30.94)	6.05 (1.57-23.73)	
Pancreas head MRI-PDFF (%)			
5.22 ± 5.67	6.24 ± 6.48	4.75 ± 5.21	.086^**^
	4.28 (0-53.76)	5.2 (0-37.21)	4.00 (0.08-53.76)	
Pancreas body MRI-PDFF (%)			
5.33 ± 5.33	6.46 ± 6.20	4.81 ± 4.81	.027^**^
	4.14 (0-43.24)	5.95 (0-32.88)	3.76 (0-43.24)	
Pancreas tail MRI-PDFF (%)			
5.20 ± 5.46	6.35 ± 6.22	4.66 ± 5.00	.032^**^
	4.10 (0-48.35)	5.9 (0-35.41)	3.43 (0-48.35)	
Pancreas MRI-PDFF (%)			
5.25 ± 5.44	6.35 ± 6.26	4.74 ± 4.95	.041^**^
	4.33 (0-48.45)	5.69 (0-35.17)	3.59 (0.02-48.45)	
L1 MRI-PDFF (%)			
46.53 ± 10.56	47.03 ± 10.29	46.29 ± 10.71	.648^*^
	47.34 (12.2-69.08)	47.71 (12.20-65.07)	47.21 (17.71-69.08)	
L2 MRI-PDFF (%)			
47.10 ± 10.59	47.44 ± 10.35	46.94 ± 10.73	.758^*^
	48.26 (11.51-71.40)	47.78 (11.51-64.79)	48.44 (16.62-71.40)	
L3 MRI-PDFF (%)			
46.92 ± 10.40	47.09 ± 9.88	46.84 ± 10.67	.877^*^
	47.43 (11.39-70.21)	47.28 (11.39-65.38)	48.03 (16.10-70.21)	
Lumbar MRI-PDFF (%)			
46.85 ± 10.38	47.18 ± 10.00	46.69 ± 10.59	.756^*^
	47.59 (11.70-69.98)	47.69 (11.70-64.15)	47.43 (16.81-69.98)	

Data are mean ± SD with median (range). *Mann-Whitney U-test, **Student’s t-test. MRI-PDFF, magnetic resonance imaging-proton density fat fraction.

## References

[1] Angulo P Nonalcoholic fatty liver disease. N Engl J Med. 2002;346(16):1221 1231. (10.1056/NEJMra011775) 11961152

[2] Neuschwander-Tetri BA Caldwell SH . Nonalcoholic steatohepatitis: summary of an AASLD single topic conference. Hepatology. 2003;37(5):1202 1219. (10.1053/jhep.2003.50193) 12717402

[3] Byrne CD Targher G . G. Nafld: a multisystem disease. J Hepatol. 2015;62(1)(suppl):S47 S64. (10.1016/j.jhep.2014.12.012) 25920090

[4] Bravo AA Sheth SG Chopra S . Liver biopsy. N Engl J Med. 2001;344(7):495 500. (10.1056/NEJM200102153440706) 11172192

[5] Caussy C Reeder SB Sirlin CB Loomba R . Noninvasive, quantitative assessment of liver fat by MRI-PDFF as an Endpoint in NASH Trials. Hepatology.2018;68 (2):763 772. 10.1002/hep.29797 PMC6054824 29356032

[6] Tang A Tan J Sun M et al. Nonalcoholic fatty liver disease: MR imaging of liver proton density fat fraction to assess hepatic steatosis. Radiology. 2013;267(2):422 431. (10.1148/radiol.12120896) 23382291 PMC3632805

[7] Idilman IS Aniktar H Idilman R et al. Hepatic steatosis: quantification by proton density fat fraction with MR imaging versus liver biopsy. Radiology. 2013;267(3):767 775. (10.1148/radiol.13121360) 23382293

[8] Idilman IS Tuzun A Savas B et al. Quantification of liver, pancreas, kidney, and vertebral body MRI-PDFF in non-alcoholic fatty liver disease. Abdom Imaging. 2015;40(6):1512 1519. (10.1007/s00261-015-0385-0) 25715922

[9] Patel NS Peterson MR Brenner DA Heba E Sirlin C Loomba R . Association between novel MRI-estimated pancreatic fat and liver histology-determined steatosis and fibrosis in non-alcoholic fatty liver disease. Aliment Pharmacol Ther. 2013;37(6):630 639. (10.1111/apt.12237) 23383649 PMC4136524

[10] Patel NS Peterson MR Lin GY et al. Insulin resistance increases MRI-estimated pancreatic fat in nonalcoholic fatty liver disease and normal controls. Gastroenterol Res Pract. 2013;2013:498296. (10.1155/2013/498296) PMC3855930 24348536

[11] Singh RG Yoon HD Wu LM Lu J Plank LD Petrov MS . Ectopic fat accumulation in the pancreas and its clinical relevance: a systematic review, meta-analysis, and meta-regression. Metabolism. 2017;69:1 13. (10.1016/j.metabol.2016.12.012) 28285638

[12] Dancey CP Reidy J . Statistics without Maths for Psychology. London: Pearson Education; 2007.

[13] Değertekin B Tozun N Demir F et al. The changing prevalence of non-alcoholic fatty liver disease (NAFLD) in Turkey in the last decade. Turk J Gastroenterol. 2021;32(3):302 312. (10.5152/tjg.2021.20062) 34160360 PMC8975521

[14] Kühn JP Meffert P Heske C et al. Prevalence of fatty liver disease and hepatic iron overload in a Northeastern German population by using quantitative MR imaging. Radiology. 2017;284(3):706 716. (10.1148/radiol.2017161228) 28481195 PMC5565690

[15] Idilman IS Ozdeniz I Karcaaltincaba M . Hepatic steatosis: etiology, patterns, and quantification. Semin Ultrasound CT MR. 2016;37(6):501 510. (10.1053/j.sult.2016.08.003) 27986169

[16] Bonekamp S Tang A Mashhood A et al. Spatial distribution of MRI-Determined hepatic proton density fat fraction in adults with nonalcoholic fatty liver disease. J Magn Reson Imaging. 2014;39(6):1525 1532. (10.1002/jmri.24321) 24987758 PMC4083476

[17] Capitan V Petit JM Aho S et al. Macroscopic heterogeneity of liver fat: an MR-based study in type-2 diabetic patients. Eur Radiol. 2012;22(10):2161 2168. (10.1007/s00330-012-2468-4) 22562090

[18] Kato S Iwasaki A Kurita Y et al. Three-dimensional analysis of pancreatic fat by fat-water magnetic resonance imaging provides detailed characterization of pancreatic steatosis with improved reproducibility. PLoS One. 2019;14(12):e0224921. (10.1371/journal.pone.0224921) PMC6886808 31790429

[19] Kühn JP Berthold F Mayerle J et al. Pancreatic steatosis demonstrated at MR imaging in the general population: clinical relevance. Radiology. 2015;276(1):129 136. (10.1148/radiol.15140446) 25658037 PMC4554208

[20] Idilman IS Low HM Gidener T et al. Association between visceral adipose tissue and non-alcoholic steatohepatitis histology in patients with known or suspected non-alcoholic fatty liver disease. J Clin Med. 2021;10(12):2565. (10.3390/jcm10122565) PMC8228492 34200525

[21] Sarma MK Saucedo A Darwin CH et al. Noninvasive assessment of abdominal adipose tissues and quantification of hepatic and pancreatic fat fractions in type 2 diabetes mellitus. Magn Reson Imaging. 2020;72:95 102. (10.1016/j.mri.2020.07.001) 32668273 PMC7442732

[22] Vieira J Amorim J Martí-Bonmatí L Alberich-Bayarri Á França M . Quantifying steatosis in the liver and pancreas with MRI in patient with chronic liver disease. Radiologia. 2020;62(3):222 228. (10.1016/j.rx.2019.11.007) 31932016

[23] Mantovani A Dauriz M Gatti D et al. Systematic review with meta-analysis: non-alcoholic fatty liver disease is associated with a history of osteoporotic fractures but not with low bone mineral density. Aliment Pharmacol Ther. 2019;49(4):375 388. (10.1111/apt.15087) 30600540

